# Fluorescence and phosphorescence lifetime imaging reveals a significant cell nuclear viscosity and refractive index changes upon DNA damage

**DOI:** 10.1038/s41598-022-26880-x

**Published:** 2023-01-09

**Authors:** Ellen Clancy, Siva Ramadurai, Sarah R. Needham, Karen Baker, Tara A. Eastwood, Julia A. Weinstein, Daniel P. Mulvihill, Stanley W. Botchway

**Affiliations:** 1 Rutherford Appleton Laboratory, Central Laser Facility,UKRI- Science and Technology Facilities Council, Harwell Science and Innovation Campus, Oxfordshire, OX11 0QX UK; 2 Rutherford Appleton Laboratory Harwell Campus, The Rosalind Franklin Institute, Didcot, OX11 0QX UK; 3 School of Biosciences, University of Kent, Canterbury, Kent, CT2 7NJ UK; 4 Department of Chemistry, Dainton Building, 13 Brook Hill, Sheffield, S3 7HF UK

**Keywords:** Biological fluorescence, Chemical biology

## Abstract

Cytoplasmic viscosity is a crucial parameter in determining rates of diffusion-limited reactions. Changes in viscosity are associated with several diseases, whilst nuclear viscosity determines gene integrity, regulation and expression. Yet how drugs including DNA-damaging agents affect viscosity is unknown. We demonstrate the use of a platinum complex, Pt[L]Cl, that localizes efficiently mostly in the nucleus as a probe for nuclear viscosity. The phosphorescence lifetime of Pt[L]Cl is sensitive to viscosity and provides an excellent tool to investigate the impact of DNA damage. We show using Fluorescence Lifetime Imaging (FLIM) that the lifetime of both green and red fluorescent proteins (FP) are also sensitive to changes in cellular viscosity and refractive index. However, Pt[L]Cl proved to be a more sensitive viscosity probe, by virtue of microsecond phosphorescence lifetime versus nanosecond fluorescence lifetime of FP, hence greater sensitivity to bimolecular reactions. DNA damage was inflicted by either a two-photon excitation, one-photon excitation microbeam and X-rays. DNA damage of live cells causes significant increase in the lifetime of either Pt[L]Cl (HeLa cells, 12.5–14.1 µs) or intracellularly expressed mCherry (HEK293 cells, 1.54–1.67 ns), but a decrease in fluorescence lifetime of GFP from 2.65 to 2.29 ns (in V15B cells). These values represent a viscosity change from 8.59 to 20.56 cP as well as significant changes in the refractive index (RI), according to independent calibration. Interestingly DNA damage localized to a submicron region following a laser microbeam induction showed a whole cell viscosity change, with those in the nucleus being greater than the cytoplasm. We also found evidence of a by-stander effect, whereby adjacent un-irradiated cells also showed nuclear viscosity change. Finally, an increase in viscosity following DNA damage was also observed in bacterial cells with an over-expressed mNeonGreen FP, evidenced by the change in its lifetime from 2.8 to 2.4 ns.

## Introduction

Viscosity is a key environmental factor within the cell, affecting numerous signaling pathways and processes. It is vital in many diffusion-mediated cellular processes, such as, metabolite diffusion, interactions between biomolecules and chemical signals, and intracellular transport^1,2^**.** Abnormal viscosity levels can result in numerous diseases for example, diabetes, Alzheimer’s disease, hypertension and arteriosclerosis^1–3^. Consequently, knowledge of intracellular viscosity changes in the course of DNA damage for example, may provide new insights in cellular biology, disease diagnosis and pathological studies and future treatment regime, and new methods of monitoring intracellular and intranuclear viscosity are needed^4^.

The standard ways to quantify viscosity of a bulk solution (rotational, capillary or falling ball viscosimeters) are not suitable for live cell studies, nor to monitor viscosity changes in real time. This barrier has been overcome by the development of a class of fluorescent probes, including so called “molecular rotors”, whose fluorescence intensity and lifetime are strongly viscosity-dependent^5^. These small donor–π–acceptor (D–π–A) structures undergo fluorescence quenching due to the formation of a twisted intramolecular charge transfer excited state after the initial light absorption: higher the viscosity, more restricted the rotation, higher the intensity, and longer the fluorescence lifetime^5–7^. Notably, emission lifetime is a more reliable parameter than the intensity, as the latter is dependent on the intensity of the excitation light source, chromophore concentration, detector calibration and many other factors. To measure local viscosity using fluorescence lifetime, a powerful technique of Fluorescence Lifetime Imaging microscopy (FLIM) is used which maps the spatial distribution of excited state lifetime of a probe, similar to that currently used for protein interactions in live cells^8^. FLIM is suitable for fluorescent molecules, i.e., those in which emission from an excited state is an allowed process that occurs within a few nanoseconds. This approach has been overwhelmingly successful in investigating a whole range of processes, from understanding the functions of plant cells, mammalian cells, viruses, materials, and diseases, to name but a few^9,10^. However, the known distinct limitations of fluorescent labels include low photostability and excited state lifetime which is of the same order of magnitude as autofluorescence emanating from intrinsic biological fluorophores. Further, if an analyte in question (oxygen, protons, ions) react with the fluorophore in a collisional, diffusion-limited process, the larger the excited state lifetime the more efficient is the quenching, and the lower the concentration of the analyte that is detectable. Phosphorescent transition metal complexes emerged a few years ago as an alternative class of labels for emission imaging, which contrary to many fluorescent labels, are photostable and have a long-lived, microsecond, emission^11,12^. In such molecules, the initially populated singlet excited state rapidly (usually sub-100 fs) and efficiently (close to 100%) transitions to a triplet excited state, which then emits a photon in a triplet-to-singlet transition back to the ground state. As this emission process is spin-forbidden, it occurs on a much longer, microsecond, time scale than the nanosecond fluorescence. The longer emission lifetime of phosphorescent probes offers much greater sensitivity to the microenvironment as interaction of the probe with the analyte occurs in diffusion-limited bimolecular reactions. The introduction of emissive metal complexes as probes has led to development of Time-resolved Emission Imaging Microscopy (TREM) and Phosphorescence Lifetime Imaging Microscopy (PLIM) which has the same principle as FLIM, but measures emission on a much longer timescale and is compatible with long-lived emissive triplet states. We previously demonstrated the use of a single instrument to achieve both FLIM and PLIM, detecting emission lifetime over six orders of magnitude (picoseconds to microseconds)^11,12^. When combined with confocal and multiphoton microscopy, FLIM-PLIM via time-correlated single photon counting (TCSPC) detection at each pixel provides an excellent spatial and temporal resolution. Thus, FLIM-PLIM-TCSPC achieves imaging over decades of time-scales, whilst preserving high spatial resolution of single and multiphoton excitation – as such, providing the wealth of data on microenvironment that is not accessible in any other single method^11,12,]^^13^.

Understanding and monitoring of nuclear viscosity is of particular importance because of the key role of the cell nucleus in storing genetic information, DNA replication and RNA transcription, gene integrity and expression. Yet little is known about any viscosity changes that may occur in the nucleus during many cell processes, partly because of lack of suitable molecular probes. Molecular rotors described above do not penetrate the nuclear membrane^14^. Green Fluorescent Protein (GFP) has been previously reported as a possible viscosity probe^15^ and probes for local RI^16–18^, and modified GFPs have been used as fluorescent sensors for intra-lysosomal viscosity^19^, but no detailed nuclear viscosity study has yet emerged.

**Figure 1:**
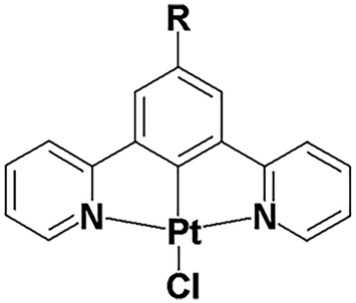
Molecular structure of the viscosity and oxygen sensitive Pt(II) complex of the cyclometalating, terdentate, N^C^N-coordinating ligand 1,3-Di(2-pyridyl)benzene, where R = H ^20,21,24^.

The present study introduces a method to determine nuclear viscosity in live cells using both FLIM and PLIM to monitor primarily changes in viscosity over the timescale from nanoseconds to tens of microseconds after induction of DNA damage. We demonstrate that changes in the lifetime of fluorescent proteins, unmodified GFP, mNeonGreen and a red FP mCherry, can be linked to intranuclear viscosity as well as RI changes. We also demonstrate for the first time, the use of a phosphorescence transition metal complex, Pt[L]Cl (L = 1,3-Di(2-pyridyl)benzene, Fig. 1), in conjunction with PLIM, as a probe for intracellular, and intra-nuclear, viscosity changes. We have previously shown that photostable Pt[L]Cl, localizes in live cell nucleus^20,21^ with little cytotoxicity on the timescale of imaging, has good two-photon absorption cross section enabling a cell-safe near-IR excitation, and has been used as oxygen sensors in cells and tumor spheroids^22,23^. We report the response of mammalian cells to damage by altering their nuclear viscosity (and RI) via radiation-induced DNA-damage. This research opens a new perspective on processes that may be affected by such changes, including gene therapy and drug action in the cytoplasm and nucleus.

## Materials and methods

### Solution phase determination of viscosity using glycerol and sucrose dilutions

All reagents, unless otherwise stated, were purchased from Thermofisher Scientific. A stock solution of GFP (a kind gift from Prof Cameron Nolan, Science and Technology Facilities Council) was prepared as 2.5 mg/ml and underwent a 1 in 10 dilution in water to obtain a final concentration of 0.25 mg/ml. Recombinant mCherry and mNeonGreen proteins were suspended in PBS to obtain a final concentration of 1.5 µM and 16 µM respectively.

Pt[L]Cl was synthesized as described previously^24^. A 10 mM stock solution of Pt[L]Cl was prepared in DMSO. A final concentration of 1 mM was prepared and used for the DMSO/glycerol preparation and sucrose/water concentrations. The stock solution was diluted in DMSO, acetonitrile or water. Viscosity measurements were performed by diluting the 1 mM solution in water-glycerol, DMSO-glycerol and sucrose-water to obtain the desired volume:volume ratio. For measurements, a drop (~ 10 µl, 100 µM) was placed on a coverslip on the Nikon Ti-E or TE2000-U microscope. Solutions were excited at 491 nm (GFP and mNeonGreen), 561 nm (mCherry) or 405 nm (3.3–20 MHz, pulse width 40 ps) (Pt[L]Cl) and emission decay kinetics collected using a Becker & Hickl SPC830 or SPC150 controlled by SPCM64^25^.

### Construction of EGFP and mCherry Ku70/80 plasmid for cell transfection

Recombinant DNAs (cDNA) of Ku70 and Ku80 were bought from Origene, Maryland, USA. The linearized vectors, pOPINE (from Oxford Protein Production Facility, UK) at 1 µg/µl were mixed with 1.0 µl of each enzyme, 1.0 µl of bovine serum albumin (BSA) at 10 mg/ml, 10 µl of 10X buffer 4, and 86 µl of MilliQ water. EGFP-Ku70 and mCherry plasmid construct was made by infusion cloning full length Ku70/80 cDNA from Ku70/80-GFPSpark (GFPSpark from Origene) into a pOPINN-EGFP (Enhanced Green Fluorescent Protein) vector provided by Protein Production UK.

### Mammalian cell culture

All cell culture reagents, unless otherwise stated, were purchased from Thermofisher Scientific. Cells were grown under humidified 5% CO_2_ in air at 37 °C and were regularly tested for mycoplasma contamination.

### Stable expressing V15B culture condition

Ku80-GFP stably expressing V15B cells (a gift from Dr. Dick van Gent) were passaged grown in phenol red free minimal essential medium (MEM) supplemented with 10% fetal bovine serum (FBS), 1% penicillin–streptomycin and 5 mM Glutamine. Cells were sub-cultured at 70–90% confluency. For imaging, cells were seeded at 5 × 10^4^ cells/well in four-well glass-bottomed chamber slides (Ibidi) and allowed to incubate for 24 h before imaging.

### Chinese Hamster Ovary (CHO) cells

Chinese Hamster Ovary (CHO) cells were purchased from ECACC (UK). Cells were grown in phenol red free DMEM/F-12 (Dulbecco’s modified Eagle Medium/Nutrient Mixture F-12) supplemented with 10% FBS, and 1% Penicillin–Streptomycin. Cells were seeded at a density of 1.2 × 10^4^ cells/well in 8-well glass-bottomed chamber slides (Ibidi). 24 h after seeding cells were transfected with Ku70/80-GFP. 200 ng of plasmid DNA per well was mixed 1:3 FuGENE HD (Promega) in Opti-MEM™ Reduced Serum Media and expression was allowed to proceed for 24 h before imaging.

### HEK293T cells transfection

Human Embryonic Kidney (HEK293-T) cell were purchased from ATCC® (USA), and cultured in phenol red free DMEM media (Gibco, UK) supplemented with 10% FBS, with non-essential amino acids, 1% penicillin–streptomycin and 2 mM Glutamine. For live-cell imaging, cells were seeded in 8 well glass bottom chamber slides (Ibidi) at concentrations of 2.5 × 10^4^ cells per well, 1 day before transfection. Cells were transfected with Fugene-HD transfection reagent (Promega, UK), as detailed by the manufacturer. 100–200 ng of DNA in 10 µl serum free, phenol red free DMEM media, mixed with 0.25 to 0.4 µl of transfection reagent and left in the tube with transfection complex for 10 min at room temperature. Then the transfection complex was added into wells containing cells, gently shaken and placed in a 37 °C incubator for at least 24 h.

### Bacterial growth conditions

Overnight cultures of *E. coli* (BL21 DE3) cells containing a mNeonGreen-fusion expression plasmid were grown at 37 °C in LB media, and used to inoculate 50 ml of TB media (Terrific broth) within shaking flasks. Media was supplemented with appropriate plasmid selection antibiotic. These cultures were subsequently grown at 37 °C with 200 rpm shaking, until OD_600_ reached 0.8–1.0, when mNeonGreen expression was induced by addition of IPTG to a final concentration of either 10 or 20 µg/ml. Samples were taken for imaging and protein analysis 2 h after 10 µg/ml induction or 5 h after 20 µg/ml induction. For cell imaging, cells were mounted on LB-agarose pads under coverslips, as described previously and imaged as described below^26^. For protein analysis, cells were pelleted at 16, 100 × RCF, resuspended in an equilibrated volume of 1 × SDS-PAGE loading, and boiled for 15 min. The samples were subsequently subjected to on SDS-PAGE gel, coomassie staining and densitometric analysis.

### Confocal and fluorescence lifetime imaging microscopy (FLIM)

Confocal images were taken using an inverted Nikon TE2000-U or Ti-E microscope attached to a Nikon EC2 scanning unit. The confocal scanning unit was equipped with a Super K Extreme NKT-SC 470–2000 nm super-continuum laser (NKT Photonics, purchased through Photonic Solutions, UK) (80 MHz repetition rate; 70 ps pulse width). Microscopy Images were acquired with a 60X (NA 1.20) water immersion objective. The excitation wavelengths were for Hoechst (405 nm excitation), GFP (491 nm excitation) or mCherry (561 nm excitation). The same wavelengths were used for one-photon FLIM. FLIM data was acquired using a Becker & Hickl TCSPC SPC830 or SPC150 module (purchased from Becker and Hickl, GmBH) equipped with a HPM100-40 detector (purchased from Becker and Hickl, GmBH) running the TCSPC software v 9.77 as described previously. We always aimed for count rates of 1E4-5E5 photons per second. Our FLIM set up can be operated as a single channel or a two-channel system via a router (HRT-41, Becker and Hickl). The filters used for mCherry and GFP were 525/39 (Thorlabs, Germany) and 633 IU filters (Comar Optics, UK) respectively. Images were taken before and after irradiation using the respective excitation laser wavelength.

### Phosphorescence lifetime imaging microscopy (PLIM)

A Nikon Ti-E microscope equipped with a Becker & Hickl 405 nm 40 ps diode laser (variable repetition rate 1–80 MHz). PLIM Images were acquired with a 60X (NA 1.20) water immersion objective. PLIM data were acquired using a Becker & Hickl DCS120 confocal unit and a TCSPC SPC150 (Becker & Hickl GmBH) module equipped with a PMC100-1 detector running the TCSPC software v 9.77. Solution phase studies where the lifetime were less than 200 ns, we found that more accurate values were obtained when the laser repetition rate was reduced to 3 MHz and the system run in a pure TCSPC single point decay mode. A 2 × 435 nm long-pass filter (provided by Becker & Hickl GmBH) was used to eliminate the excitation wavelength. Cells were labelled with 100 µM Pt[L]Cl (similar to those used in the solution phase and calibration studies) prior to imaging and incubated at 37 °C for 20 min or more. Images were taken before and after inducing the DNA damage.

### One-Photon 405 nm Irradiation induction of DNA damage

Hoechst 33258 (Sigma Merck) was added to the cells, at a concentration of 160 µM, and incubated at 37 °C for 20 min. FLIM/PLIM images of cells were taken before and after irradiation using the respective excitation laser wavelength above. The confocal software EZC1 Version 3.91 was used to generate line illumination (using the line function with a pixel dwell time of 5 µs repeated for 10 s) across either the nuclei alone or nuclei and cytoplasm of individual cells (few µW power) with 405 nm (BSM-L, Becker and Hickl, Germany) for one photon excitation.

### Multi-Photon 730 nm Irradiation induction of DNA damage

Multiphoton laser irradiation was performed using a modified Nikon EC2 confocal scanhead^25^ Similar to the one-photon excitation, Hoechst 33258 was added to the cells, at a concentration of 80 µM, and incubated at 37 °C for 20 min. FLIM and PLIM images were acquired before and after laser irradiation using 730 nm from a Coherent Mira 900F (pumped by Verdi 532 nm CW laser, Coherent Laser, UK), 76 MHz, 180 fs tuned to 730 nm wavelength. A line across the nuclei of cells was irradiated in the EZ-C1 confocal software using an approximately 5mW at the sample, similar to those we have used previously^27^. Initial system calibration and laser power measurement at the sample were performed by replacing the microscope condenser with an identical 60 × water objective. The laser power out of the second objective divided by the transmission factor (at 730 nm through the objectives) from the manufacturer website, gave the average laser power at the sample.

### X-ray irradiation for induction of cellular DNA damage

Cells were irradiated with 25, 50 and 75 Gy hard x-rays at a dose rate of 1.7 Gy/min at the UK Health Security Agency. The X-ray source was regularly calibrated by the manufacturer. The X-ray source used was a 250 kVp, 13.0 mA at 500 mGy/min. (AGO X-Ray Ltd., West Coker, UK) fitted with both 1 mm copper and 1 mm aluminum filtering.

Irradiations were performed at 4 °C to minimise DNA repair processes.

Following irradiations, cells were delivered to the culture laboratory (on ice) and incubated for either 20 min, 30 min and 5 h at 37 °C with humidified 5% CO_2_ in air before FLIM/PLIM imaging or fixing using cold methanol for 20 min.

### FLIM-PLIM data analysis

FLIM and PLIM images were acquired at 256 × 256 pixels with each pixel containing a full decay profile. The data were analysed using Becker&Hickl SPCImage V6.4 or V.7.1. During the data analysis, only photon count of more than 1000 or more than 100 counts in the first signal channel was deemed good enough for significant decay statistics. In the SPCImage software, it is necessary to discard pixels with poor photon signal-to-noise ratio and by adjusting the threshold range appropriately. Emission decay kinetics are analysed as mono- or multi-exponential functions. Majority of our analysis (for FLIM) used the incomplete multi-exponential function with a laser repetition rate of 12.5 ns. This accounted for the fact that the NKT laser used in this work operate at ~ 80 MHz which could not be varied. The fitting algorithm model used was the maximum likelihood estimation. Although in our work, this was found to be similar to the weighted least square model.

We regularly measure our instrument response function (IRF) using very dilute ludox solution which is around 80 ps. The SPCImage software (all versions) also generates very accurate IRF to calculate the lifetime. Indeed it has been deduced that this method of estimating the IRF is perhaps better than the measured value. Our ludox method and software calculated are generally in agreement. We refer the reader to the technical note by Wolgang Becker (Becker and Hickl), https://www.becker-hickl.com/modelling-of-irf-makes-irf-recording-unnecessary. Chi-square (χ^2^) is used to determine the goodness of the fit. Chi-square (> 1.3) denotes presence of multiple fluorophore components and (< 0.8) represents poor fit of the data point. The mean lifetime from at least three experimental repeats of emission decay of GFP, mNeonGreen, mCherry or Pt[L]Cl in water or DMSO /glycerol mixtures in the viscosity range from 1 to 250 cP was calculated and used as calibration. The data were fitted as a semi-log viscosity against lifetime values.

## Results

### Solution phase characterization of fluorescence lifetimes of fluorescent proteins in glycerol:water mixtures

**Figure 2:**
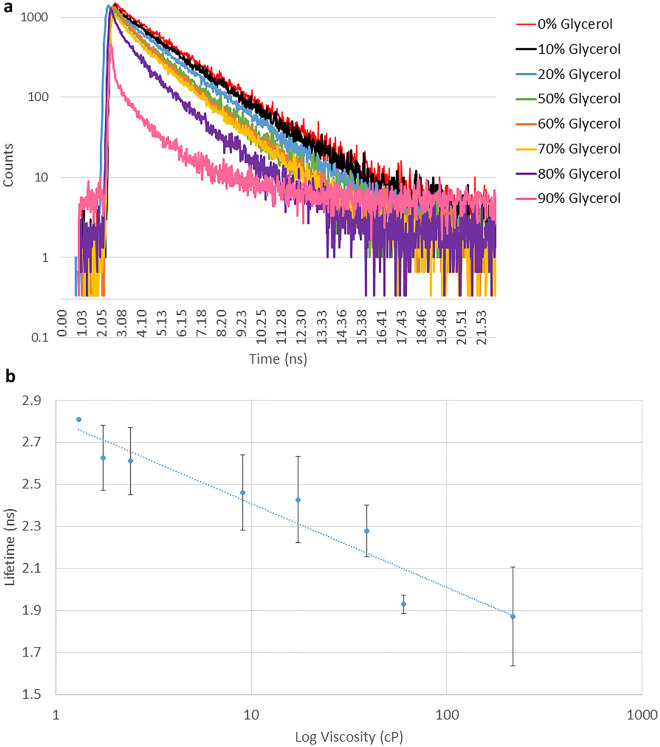
(**a**) Viscosity dependence of 0.25 µg/ml GFP fluorescence lifetime in solution at various glycerol concentrations. Semi-logarithmic plot of the fluorescence lifetime as a function of glycerol/water ratio. The fluorescence decay data shown are representative of three measurements. (**b**) Viscosity sensitivity of GFP with fluorescence lifetime displayed as centipoise (cP) in varying glycerol / water concentration. Standard deviation plotted as error bars.

Figure 2a shows the fluorescence decay kinetics of GFP in varying glycerol/water mixtures following excitation at 491 nm, emission was collected at 520 ± 35 nm. The change in the composition of glycerol:water mixture does not affect the excitation and emission maxima^28^. A single exponential decay of GFP fluorescence was observed at glycerol concentrations less than 80% with χ^2^ between 0.9 to 1.3. At glycerol concentrations > 85%, a multiexponential decay was observed, as also reported previously^18^ indicating a presence of multiple microenvironments, and hence not included in the linear plot for the analysis. Figure 2b illustrates the linear relationship between the fluorescence lifetime of GFP and log(viscosity); the glycerol percentage was expressed as viscosity (cP)^29^. The addition of glycerol caused the lifetime of GFP to decrease from 2.63 (1.74 cP) to 1.87 ns (219 cP), which is in agreement with previous EGFP viscosity and refractive index studies^17,28^. Additionally, lifetime of GFP crystals measured as low as 1.66 ns (Fig. S1). Surprisingly, mCherry in solution showed an increase in lifetime from 1.50 to 1.93 (± 0.2) ns with increasing viscosity (1.76–1270 cP) (Fig. S2) while mNeonGreen showed a decrease in lifetime from 2.89 to 2.25 ± 0.1 ns (Fig. S3). It is worth noting that mNeonGreen has a lifetime of 3.1 ns in solution containing dissolved ions such as phosphate buffer saline but only 2.91 ns in water alone. The response of these two modified fluorescent proteins to viscosity or RI have not been reported. Varying the temperature of the GFP-glycerol/water environment resulted in some change in lifetime of the fluorescent proteins below 35 °C, with a large decrease in lifetime observed above this temperature (Fig. S4), potentially caused by increased non-radiative decay at higher temperatures^30^ although a decrease in viscosity as a contributing factor cannot be ruled out. From the data presented here, we tentatively conclude that GFP likely responds not only to changes in the RI (as shown in Fig. 2b) but also some viscosity change. Indeed Suhling et al. have shown a clear viscosity dependence of GFP lifetime in a cellular environment and bathed in glycerol^31^.

### Pt[L]Cl phosphorescence lifetime in glycerol:water and sucrose:water mixtures

**Figure 3:**
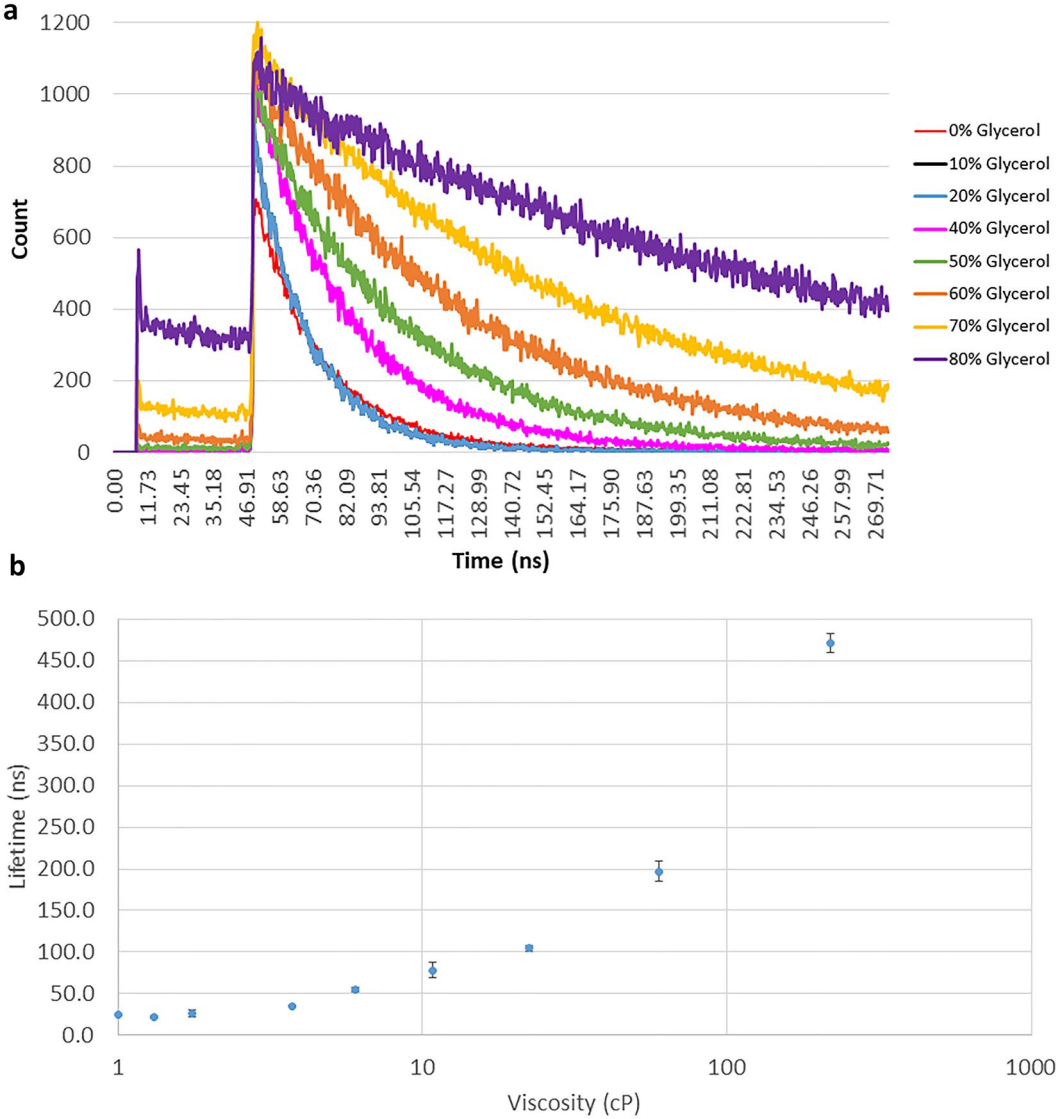
(**a**) Viscosity dependence of Pt[L]Cl phosphorescence lifetime in solution with varying glycerol/water ratio (stock solution of 1 mM of Pt[L]Cl in DMSO was used to reach the final concentration of Pt[L]Cl of 50–100 µM). Plot of the phosphorescence decay of Pt[L]Cl shown are representative of three measurements. (**b**) Viscosity dependence of Pt[L]Cl phosphorescence lifetime in solution with varying glycerol/water ratio. Stock solution of Pt[L]Cl, 1 mM in DMSO, was used before dilution to the final concentration of 50–100 µM. Standard deviation plotted as error bars (note: some errors are smaller than the symbols).

Figure 3a shows the phosphorescence decay kinetics of Pt[L]Cl in glycerol:water mixtures (prepared from a DMSO 1 mM stock solution of Pt[L]Cl to the concentration of 50–100 µM). An increase in the phosphorescence lifetime from 220 to 470 ns was observed with an increase in glycerol concentration corresponding to viscosity changes from 1.31 to 219 cP. A semi-log/log plot of the phosphorescence lifetime versus glycerol concentration shows a non-linear relationship (Fig. 3b). The emission decay was satisfactorily fitted with a mono-exponential function, indicating that emission originated from one type of species. Little or no change in Pt[L]Cl lifetime was observed when the temperature was increased from 5 to 50 °C (Fig. S5).

**Figure 4:**
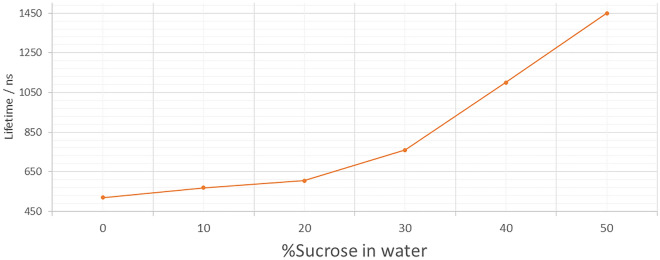
Viscosity dependence of Pt[L]Cl (50–100 µM) phosphorescence lifetime in water with varying sucrose concentration. Error margin is with 10% standard deviation.

Aqueous solutions of sucrose of a range of concentrations were prepared to further determine viscosity effect on emission lifetime of the probes. A stock solution of Pt[L]Cl in acetonitrile was used for the viscosity studies in acetonitrile/sucrose:water (aq) mixtures. Figure 4 shows an increase in the phosphorescence lifetime of the probe with increasing sucrose percentage, from 520 ns (acetonitrile alone) to 1.45 µs sucrose:water (50%), equivalent to viscosity changes from 1.3 to ~ 200 cP. Since the lifetime of Pt[L]Cl is known to be oxygen-sensitive, we purged the sucrose:water (50%) mixture using argon for up to 30 min. The phosphorescence lifetime increased further upon purging from 520 ns to 2 µs for acetonitrile alone and 7.4 µs for acetonitrile-sucrose:water (50%) (Fig. S6). Thus the phosphorescence lifetime of Pt[L]Cl in the absence of oxygen increases with respect to increase in viscosity. Persistent illumination (several minutes) of Pt[L]Cl in solution alone does not lead to a phosphorescence lifetime change.

### FLIM of GFP and mCherry expressed in mammalian cells and within the nucleus

#### One-photon 405 nm irradiation of live cells

**Figure 5:**
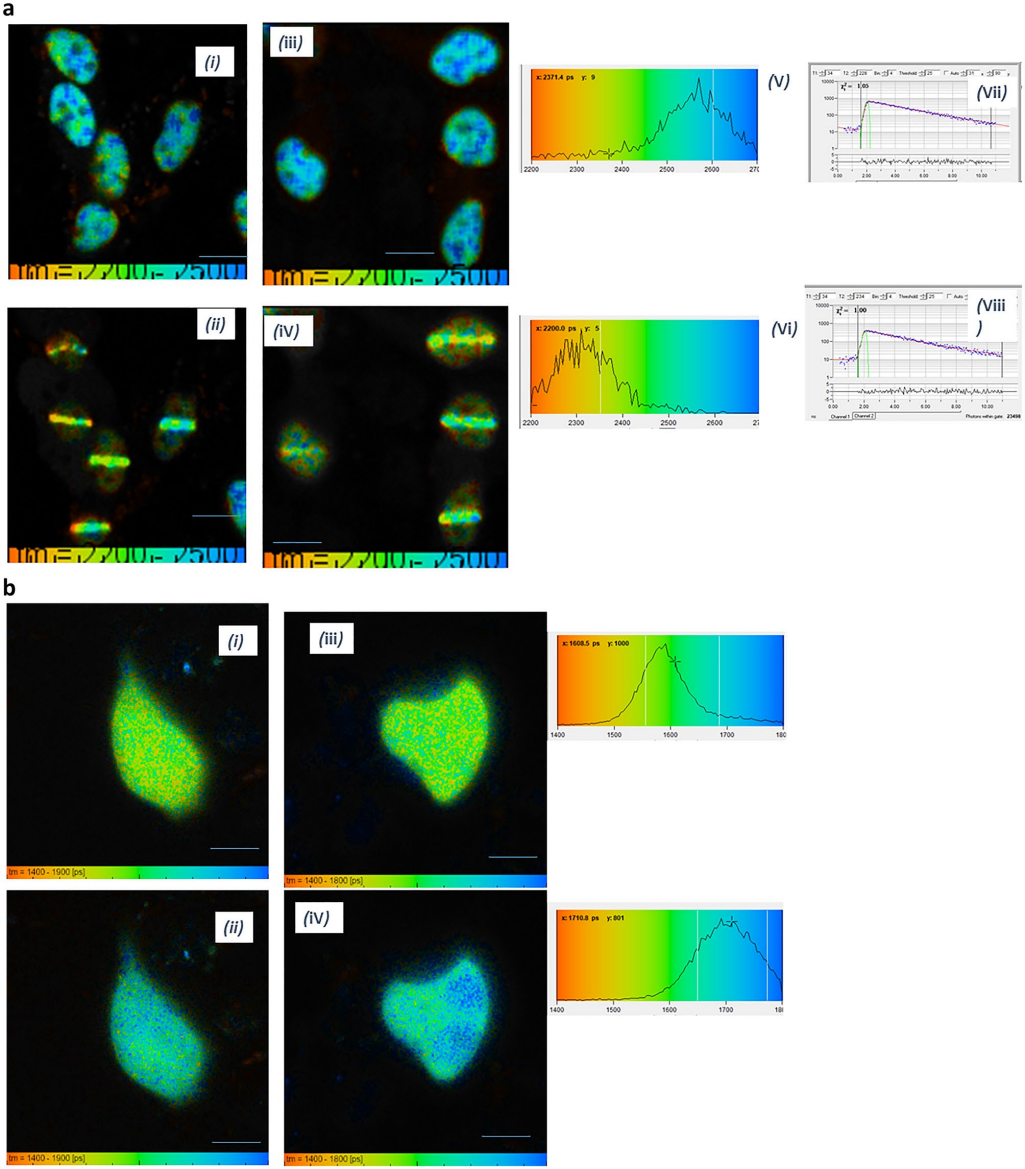
(**a**) Viscosity change in V15B mammalian cells expressing DNA repair protein GFP-Ku80. FLIM of cells before laser microbeam irradiation (i and iii). FLIM after microwatt (~ 1 µW average power) irradiation (ii and iv). Histograms for i and ii (v and vi) shows the shift in GFP lifetime indicating significant viscosity change. vii and viii typical decays. Scale bar 10 µm. (**b**) Viscosity change in CHO mammalian cells expressing non-specific mCherry fluorescent protein. FLIM of cells before laser microbeam ~ 1 µW irradiation (i and iii). FLIM after microwatts irradiation (ii and iv). Histograms shows the shift in mCherry lifetime indicating significant viscosity change. Scale bar 10 µm.

Confocal and FLIM microscopy of V15B cells show that GFP fused to the DNA double strand break repair Ku80 localizes mostly to the nucleus and accumulates at double strand breaks (DSBs) following 405 nm laser micro-irradiation in the presence of Hoechst dye (Fig. 5a). This is consistent with previously published data^32,32,34–36^. The GFP-Ku80 lifetime determined via FLIM showed a change in the whole nuclear lifetime from 2.5 ± 0.07 ns to 2.29 ± 0.07 ns following 10 secs of µW levels of 405 nm irradiation (see methods section for laser irradiation conditions). The excited state lifetime change alone corresponds to a change in viscosity from 3 to 20.56 cP within the nuclei. The lifetime of the irradiated line decreases to approximately 2.28 ± 0.07 ns while the surrounding area of the nuclei decreases to approximately 2.35 ± 0.06 ns. 5 s of µW irradiation also gave a lifetime change from 2.40 ± 0.08 to 2.30 ± 0.07 ns and indicated an increase in viscosity from 10.57 cP to 18.42 cP. Similarly, the whole nuclear lifetime decreased, with the biggest lifetime change at the irradiated line. Additionally, the lifetime of non-specific GFP expressed in cells decreased from 2.53 ± 0.1 ns to 2.45 ± 0.6 ns in HEK293 cells following 10 secs of µW irradiation.

Conversely, the lifetime of non-specific mCherry transfected in CHO cells increased from 1.55 ± 0.07 ns to 1. 7 ± 0.07 ns in CHO cells following 3 s of ~ 1 µW 405 nm laser irradiation (Fig. 5b).

#### Multi-photon 730 nm irradiation to induce DNA damage of live cells

**Figure 6:**
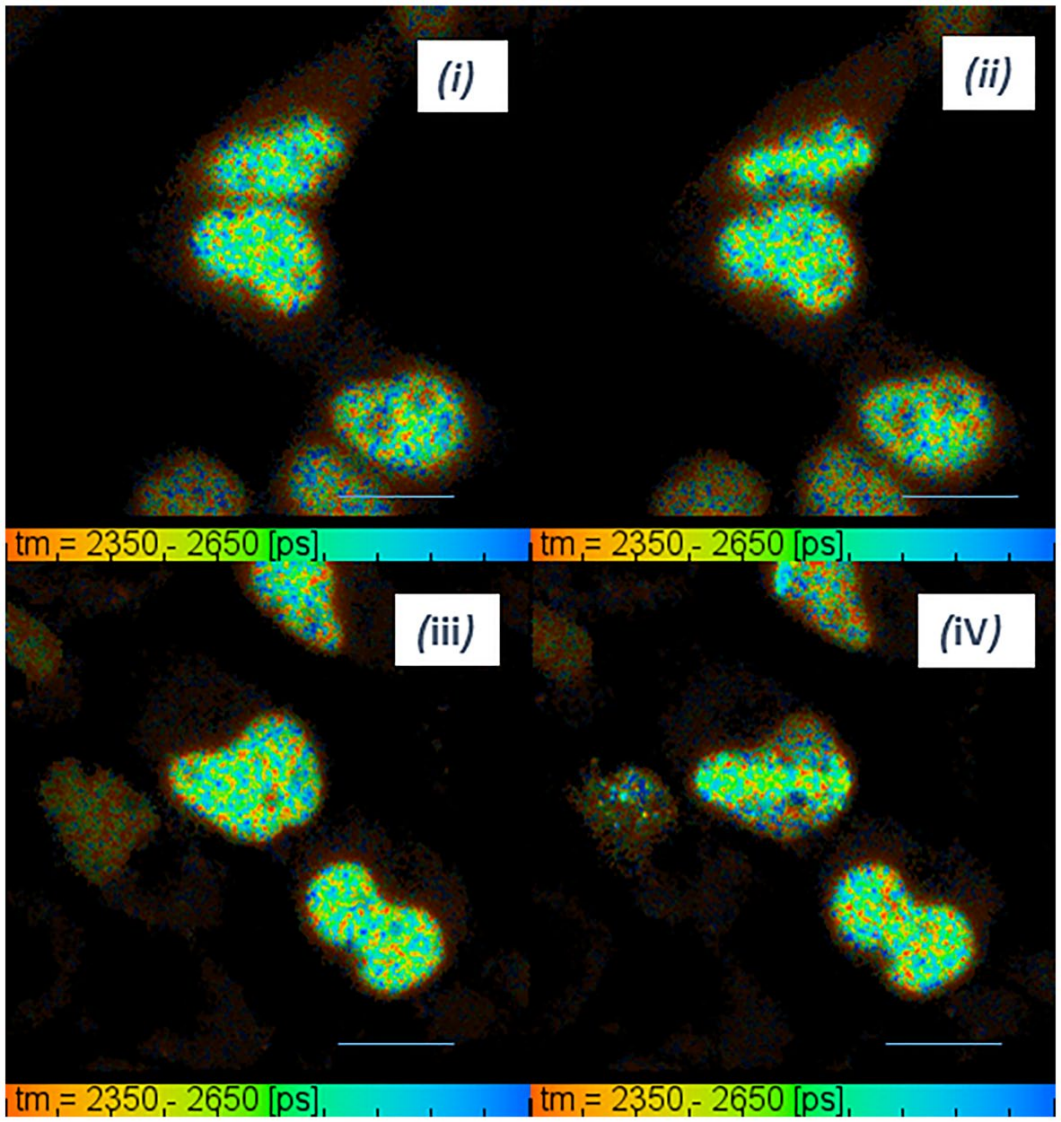
Multiphoton excitation at 730 nm, (5 mW average power), shows small viscosity change in V15B mammalian cells expressing Ku80-GFP fluorescent protein. FLIM of cells before laser microbeam irradiation (i and iii). FLIM after milliwatts irradiation (ii and iv). Only small shift in GFP lifetime indicating some viscosity change unlike the one-photon excitation. Scale bar 10 µm.

Multiphoton irradiation showed Ku80-GFP recruitment to the DNA damage site (Fig. 6). However contrary to 405 nm irradiation, only a small fluorescence lifetime change was seen, from 2.55 ± 0.7 before irradiation to approximately 2.45 ± 0.7 ns at the microbeam irradiated line. No lifetime changes were observed throughout the rest of the nuclei as it remained at 2.55 ± 0.6 ns. We speculate that as well as cells responding to the DNA damage (in the one-photon process), photo-toxicity may also contributes to the viscosity change, with the near infra-red light being significantly less toxic than UV/visible light.

### DNA damage by one-photon laser irradiation of mammalian cells incubated with Pt[L]Cl that localized in nucleus and cytoplasm

**Figure 7:**
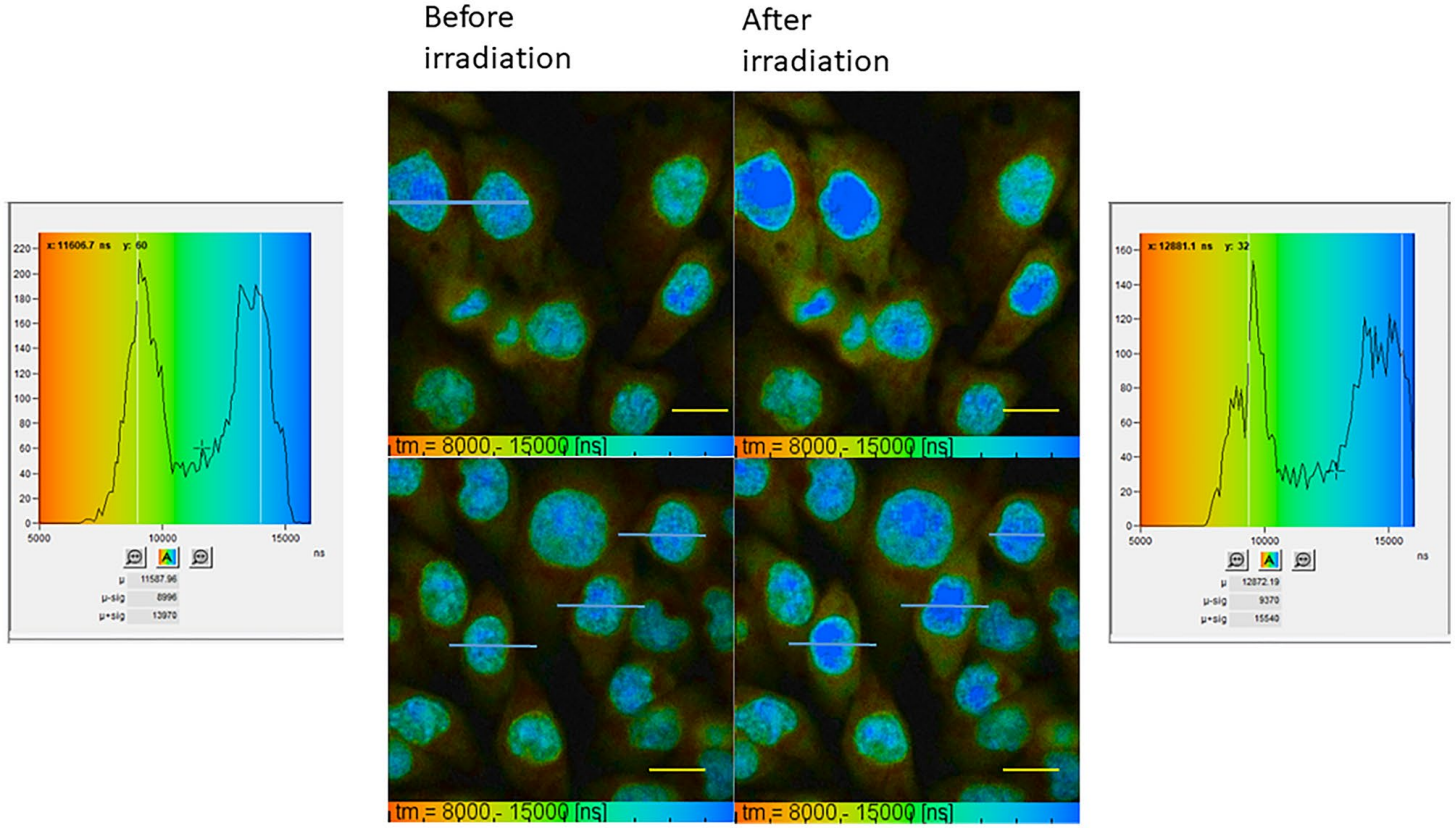
Viscosity changes in CHO cells labelled with Pt[L]Cl. PLIM map of cells before 405 nm laser irradiation (i and ii). PLIM after microwatts irradiation (ii and iv). Blue lines indicate irradiated cells to guide the eye. Scale bar 10 µm.

PLIM of irradiated cells showed an increase from 12.5 ± 0.8 to 14.1 ± 1.1 µs in the lifetime of Pt[L]Cl located in the nucleus of the CHO cells (co-labelled with Hoechst) following 10 s of µW 405 nm laser irradiation (Fig. 7). The lifetime of the Pt[L]Cl in the cytoplasm increased from 8.5 ± 0.3 to 9.2 ± 0.7 µs. This increase in lifetime corresponds to an increase in viscosity following DNA damage (see Fig. 2b), supporting the findings of GFP viscosity studies. The level of lifetime change was also found to be sensitive to the amount of possible DNA damage. For example, laser micro-irradiation for 20 secs (~ 1 µW compared with 10 secs resulted in a 1.84 µs increase in nuclei lifetime (11.9 ± 0.8 to 13.7 ± 1.3 µs) while 30 s of repeated irradiation (scanning) resulted in a 1.53 µs decrease in nuclei lifetime (11.3 ± 0.3 to 9.8 ± 0.3 µs) at the damage site. It was also observed that at a higher irradiation conditions, the cell damage was too high for the cell to respond in a ‘natural’ manner and little or no lifetime change was observed. Furthermore, fluorescence signal bleaching occurred above 30 s laser irradiation with ~ 1 µW irradiation at 405 nm. Unlike Ku80-GFP labelled cells, no recruitment or fluorescence accumulation was seen at the damage site when the Pt[L]Cl alone (no Hoechst) was used. This was as expected as the probe was not tagged to any DNA repair protein (non-specific labelling) and therefore found throughout both the cytoplasm and nucleus. Interestingly, neighboring cells also showed a change in lifetime and thus a viscosity increase following irradiation. An average increase in nuclear lifetime of 1.53 µs (from 12.05 to 13.58 µs) in CHO cells labelled with Hoechst and Pt[L]Cl was observed after a neighbor cell was exposed to 405 nm laser irradiation. It is thought that Pt[L]Cl is more sensitive to cell environmental changes compared to GFP or mCherry.

To confirm that the lifetime changes are a result of cell damage (through phototoxicity and DNA damage), mammalian cells expressing GFP-Ku80 were fixed using cold methanol. Upon 405 nm or 488 nm laser damage, the GFP lifetime did not change and remained at 2.6 ns (Fig. S7). Similarly persistent illumination of GFP in solution alone does not lead to a fluorescence lifetime change, except the process of photo-bleaching.

### Determination of viscosity change during X-ray irradiation of mammalian cells

FLIM of V15B cells showed a modest change in GFP lifetime following x-ray irradiation from approximately 2.50 ns to 2.45 ns and 2.40 ns at a dose of 25 Gy and 50 Gy respectively. PLIM showed an increase in the lifetime of Pt[L]Cl in the nucleus of CHO cells from approximately 6.5–8 µs to 8–10.5 µs following 25 and 50 Gy X-ray irradiation. The phosphorescence lifetime of Pt[L]Cl in the cytoplasm increased from 3.5–5 to 4–6 µs, again indicating an effect of ionizing radiation on the viscosity of cells. The small reduction in lifetime of the GFP following ionizing radiation is possibly due to the nature of interaction of ionizing radiation with matter: simulations show that majority of the interaction only occurs at the low energy transfer (LET) of X-ray ‘track’ ends. It has been demonstrated that 25–50 Gy possibly deposits only few radiation tracks within the cell volume^34^. Assuming low LET radiation generates only a few DNA double strand breaks per Gy, the dose used here may not be sufficient to significantly alter the environment of the cell. Methods to increase the sensitivity here would be highly beneficial. On the other hand, a laser microbeam irradiation deposits significant amount of energy leading to an equivalent high LET radiation characteristics to cause a large viscosity change^37^.

### Abnormal physiology in cells causes cell viscosity and / or RI change

**Figure 8:**
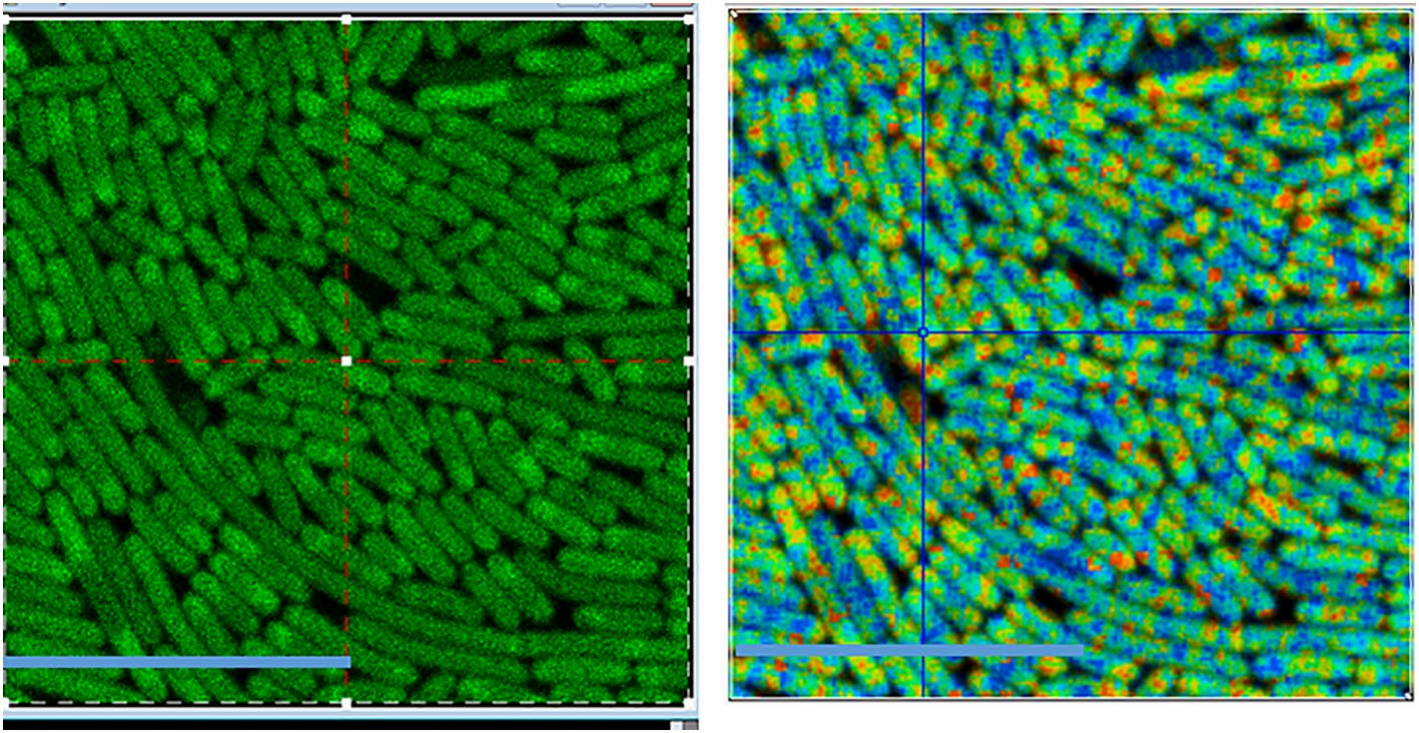
*E. coli* expressing VNp2-mNeonGreen fusion protein at differing protein concentrations. Left: confocal image. Right: FLIM map. A decrease in the mNeonGreen lifetime from 2.8 to 2.4 ns is seen in higher expressing cells, indicating significant viscosity change. Scale bar 10 µm.

We have observed in both mammalian and bacteria cell a significant fluorescence lifetime change in the cells overexpressing a fluorescent tagged protein. For example, mammalian cells expressing GFP-Ku80 shows a range of FP fluorescence lifetimes of 2.2–2.7 ns. This wide range is attributed to environmental changes in the cell possibly caused by a change in viscosity due to the increased protein production. It is worth noting that increased protein concentration in cells may also lead to RI change^38^. Similarly, using an inducible bacterial expression system, we have observed that a sevenfold increase in mNeonGreen expression is sufficient to reduce the lifetime of the fluorescent protein from 2.8 to 2.4 ns (Fig. 8). This is equivalent to a change from 5 to > 100 cP.

## Discussion

The organization of content during cellular processes such as DNA damage and repair and during drug action and transcription is still largely unknown. Moreover, microscopy at subcellular resolution has shown formation of structures and entities such as foci, droplets, aggregates and granules, functions of which cannot be currently explained. The mechanism of formation of these structures are also unknown. We have previously shown that the cell cytoplasm viscosity changes in the presence of cytotoxic agents such as reactive oxygen species, specifically hydroxyl radicals^6^. The use of molecular rotors, to detect effective cell cytoplasmic viscosity cannot be readily employed for the nucleus of live cells, although studies are underway to remedy this limitation^39^. Considering the importance of the cell nucleus and the changes that constantly occur, such as replication, transcription and DNA damage, we sought to discover new fluorescent probes for nuclear environment phase and viscosity change to further understand changes in the nucleus. Here we test and report two emissive probes, fluorescence proteins and phosphorescent Pt[L]Cl for determining changes in nuclear viscosity.

We show that Pt[L]Cl previously used for oxygen sensing can now be used for monitoring viscosity. The phosphorescence lifetime of the Pt[L]Cl probe in varying DMSO/glycerol, water/glycerol or sucrose/water concentrations (Fig. 3a) changes by around twenty times (20–470 ns), and further to over a microsecond in live cells. This is several orders of magnitude higher than the nanosecond changes of the current bodipy-based molecular rotors or GFP used to monitor changes in cell cytoplasmic viscosity and RI. The phosphorescence lifetime of solutions of Pt[L]Cl prepared from a stock solution in acetonitrile diluted in water/glycerol mixtures, and in sucrose/water solutions, were found to increase with the increase of glycerol or sucrose concentration (Fig. 4b and Fig. S6). These data were used to produce a calibration curve of Pt[L]Cl lifetime as a function of viscosity. Then, the changes in phosphorescence lifetime of Pt[L]Cl in mammalian cells can be related to changes in viscosity. Since emission properties of Pt[L]Cl are also sensitive to oxygen concentration, purging a solution of Pt[L]Cl in sucrose/water with argon showed further increase in the phosphorescence lifetime, indicating a response to both oxygen and viscosity, Fig. S6. Importantly, there are no reports of oxygen concentration changes in cell nucleus upon DNA damage. We therefore conclude that the changes in Pt[L]Cl phosphorescence lifetime observed here are likely due to changes in cellular viscosity. Cell cytoplasmic viscosity change upon cell damage has been previously reported but not for the cell nucleus^6^.

In this study, we also used GFP (and its derivatives mNeonGreen and mCherry) and Pt[L]Cl to examine possible changes in nuclear viscosity and to a lesser extend RI, following DNA damage induced by either one-photon laser irradiation (405 nm), multiphoton irradiation (730 nm, in the presence of Hoechst dye), or X-ray irradiation. Ionizing radiation such as x-rays can cause damage directly to the DNA bases, or indirectly through formation of reactive oxygen species^40^. Large amounts of DNA single strand breaks, base damage and lower yields of double-strand breaks (DSB, ~ 30/Gy) have been reported after irradiation^41^. Ionizing radiations mostly damages the DNA bases and the helix, including chromosome aberrations. However, non-specific effects such as bystander and genomic instability have been observed^42^. Low-LET ionizing radiation induces on average 20–40 DSBs per Gy in different cell types, increasing in the presence of oxygen or with high-LET irradiation. High-LET radiation such as those from alpha-particles, protons and carbons ions produces complex clustered sites of DNA damage which are harder to repair^43^. The relationship between the better outcome from proton therapy for patients and that of cellular environment changes are still unclear and needs further studies. Similarly, UV irradiation causes DNA damage both directly and indirectly. UVB (280–320 nm) and UVC (190–290 nm) mostly cause direct damage to DNA bases, while UVA (320–400 nm) interacts with photosensitisers to induce DNA damage^44–46^. The major lesions produced by UV-excitation are 8-oxodGuo, cyclobutene pyrimidine dimers (CPDs) and (6-4) pyrimidine photoproducts (6-4-PPs, those are not produced with UVA). Photosensitizers such as Hoechst 33,258 and 33,342 have been used in combination with UVA irradiation to induce DSBs and increase DNA damage^44,45^. Tenfold higher 405 nm laser intensity was required in the absence of Hoechst 33342^46^ to achieve the same level of damage.

Lifetime imaging revealed that irradiation of mammalian cells for 10 secs at 405 nm (1 µW or less) causes a decrease in GFP lifetime from 2.55 ± 0.05 to 2.29 ± 0.07 ns, but an increase in the lifetime of nuclei-localized Pt[L]Cl from 12.6 to 14.2 µs. Similarly, a decrease in GFP lifetime from approximately 2.50 ns to 2.40 ns and an increase in the phosphorescence lifetime of nucleus-localized Pt[L]Cl from 6.5–8 µs to 8–10.5 µs following 50 Gy and 25 Gy x-ray irradiation respectively, was observed. Whilst a reduction in GFP lifetime upon an increase in concentration of macromolecules has been reported^38^, it is clear that the changes we observed are not due to protein concentration effect, as our studies show a reduction in protein lifetime across the cell upon irradiation. An increase in the phosphorescence lifetime of Pt[L]Cl localized in the cytoplasm was also observed following both UVA (with Hoechst) and x-ray irradiation. Interestingly the lifetime of mCherry increased in the cell nucleus upon UV irradiation in line with that seen in solution phase studies. All these observations demonstrate an increase in cellular nuclear viscosity following DNA damage. A change in entire nuclear viscosity was seen following 405 nm irradiation, whilst when DNA damage was induced by multiphoton irradiation (730 nm with Hoechst), only the damage site showed a viscosity change. This difference is as expected as femtosecond multiphoton irradiation allows more precise localization of damage than ionizing radiation or one-photon UV damage. In this studies, the femtolitre volume of DNA damage generated by the multiphoton process compared to the one photon process is expected to lead to difference in cellular response. The NIR light is not absorb throughout the excitation cone from the focusing microscope objective. Whilst the one-photon process at 405 nm would highly likely cause other reactive oxygen species (ROS) generation as well as DNA double-strand breaks (DSBs) across the large focus cone in the z-plane. The viscosity change may be due to the recruitment of several repair proteins to the damage site. Both ionizing radiation and laser induced DNA DSB generates foci of γH2AX, phosphorylation of the H2A.X histone. The reason for the foci formation is unknown. Further, the use of super resolution microscopy techniques have shown that the foci are as small as 50 nm or less, clustered in groups as many as eight or more^47^. The importance of such foci structure is also unknown, and how this contributes to the change in viscosity around the damage site needs further investigation. We speculate that the change in nuclear viscosity may aid in localizing these membrane-less structures. The formation of such biological entities, at times described as ‘biomolecular condensates’ has been observed in a number of cell processes other than DNA damage^20,21,48–51^. However, the cellular function of these condensates with high concentration of nucleic acid and proteins are still under debate (discussed further below).

Previous studies have elucidated an increase in cytoplasmic intracellular viscosity following laser irradiation. The ratiometric approach to study the response of a conjugated porphyrin dimer to viscosity has so far been very useful for determining cytoplasmic viscosity. Blue laser irradiation perturbed single cells and resulted in a large increase in intracellular viscosity due to the production of singlet oxygen^6^. Furthermore, an increase in cytoplasmic viscosity following photobleaching of 53BP1-GFP U2OS cells was observed^48^. Our results also support the notion of an increase in viscosity upon cell damage. In addition to an increase in whole nuclear viscosity in the damaged cell using the novel Pt[L]Cl probe, we observed that neighboring cells also showed lifetime and viscosity changes (Fig. 6). For example, in CHO cells labelled with Hoechst and Pt[L]Cl, phosphorescence lifetime of Pt[L]Cl increased up to 2.54 µs when neighboring cells were exposed to 405 nm irradiation. This change is believed to occur due to the bystander effect, however, the mechanism for this process is unknown and would be the subject for future studies.

The increase in viscosity in particular (and RI) following irradiation may also be due to a number of other reasons. The fast diffusion controlled recruitment of several DNA damage repair factors to DSBs such as Ku, DNA ligase IV and MRN complex may increase nuclear viscosity through macromolecular crowding^34,50–53^. Additionally, ionizing radiation results in chromatin remodeling which initiates the recruitment of these DNA repair proteins to ionizing radiation-induced foci^54^. Such chromatin rearrangements may cause viscosity and RI changes. Alternatively, actin filaments visualized within the nucleus play a role in the organization and transport of nuclear contents as well as other key processes such as transcription, regulation and chromatin structure^55^. Recent studies report nuclear actin polymerization as part of the DNA damage response providing a possible explanation for the increase in viscosity seen upon laser irradiation^54^. It has been revealed that polymerized actin binds to Ku, potentially regulating non-homologous end-joining, the first defense mechanism of DNA DSBs that is cell-cycle independent. However, it was later revealed that spin-down assays are not a good indicator of nuclear actin affinity, and further work is needed to clarify the interactions between nuclear actin and repair proteins^55^. Furthermore, increased DNA DSBs in vitro and in vivo were observed following actin disruption via drug treatment, suggesting reduced DSB repair efficiency or increased DSB frequency^55^. As mentioned above, recent studies have also revealed biomolecular condensate formation upon DNA damage and repair. Biomolecular condensates are membrane-less, intracellular compartments that concentrate biological molecules^56^. DNA strand break foci may be acting to contain the damage or may be acting as an effective signalling process to the repair mechanism in a viscous environment.

There is limited published research on the use of fluorescent probes for determining nuclear viscosity. A two-photon fluorescent probe, TP-2Bz that targets the nucleus, described as a ratiometric viscosity probe was recently reported^57^. TP-2Bz in the nucleus increased emission intensities at 407 and 650 nm suggested increased viscosity. The use of increase in intensity alone as a measure of viscosity change may be prone to errors such as described above. Furthermore, in 2020 Yu et al*.* developed V-P1, a fluorescent probe which can be used to determine viscosity changes through fluorescence intensity and fluorescence lifetime in both the cytoplasm and nucleus. The effects of a range of stimuli such as monensin treatment and temperature on viscosity were studied^58^. However, neither of these probes were used to study viscosity changes following phototoxicity and DNA damage, future work is therefore needed to investigate the response of these fluorescent probes after laser and x-ray irradiation.

Previous work on the fluorescence decay of EGFP revealed different exponential fits were required depending on the laser excitation wavelength. A three exponential fit has been used when GFP was excited at 400 nm, 420 nm or 440 nm, a biexponential fit has been used at 460 nm or 488 nm excitation and a monoexponential fit has been used at 543 nm excitation^59^. Our results show EGFP to have a biexponential decay at increasing viscosities significantly above that of water. Complex decay profile was observed in viscous solutions (> 85% glycerol) similarly to that observed by Suhling et al.^28^. This is believed to occur due to protein denaturing of the β-barrel structure of GFP. Alternatively, the viscosity of the solution may influence the form of GFP (protonated versus deprotonated), thus influencing the lifetime. In this study, we developed and proposed the use of GFP alone as a viscosity as well as RI sensing probe in both cell cytoplasm and the nucleus when combine with FLIM. Previous reports have used modified GFP as a viscosity reporter. For example, in 2018^19^, synthesized Lys-V, a fluorescent probe based on *p*-hydroxybenzylideneimidazolidinone (HBDI), the fluorophore responsible for fluorescence in GFP. Increasing viscosity showed an increase in fluorescence intensity within lysosomes^19^. However, this is the first application of unmodified GFP protein as a viscosity (as well as RI) sensing probe.

### Differential yield of protein production leads to cytoplasmic viscosity change

Having confirmed GFP and its derivatives capable of reporting cellular viscosity, we next investigated the effect of protein over-expression using bacterial cells. We observed protein expression yields of 0.15 mg mNeonGreen/ml of culture (low yield) and 1.08 mg mNeonGreen/ml of culture (high expression). These levels are sufficient to alter the mNeonGreen lifetime between 2.4 and 2.7 ns. This lifetime is significantly smaller than that of mNeonGreen in solution (2.8–3.1 ns) and in exponentially growing mammalian cells, indicating a significant change of possible cellular viscosity (10–100 cP), based on the decrease in lifetime of mNeonGreen in the glycerol/water mixtures.

A modest change in the GFP lifetime following x-ray irradiation of cells leading to DNA damage (at room temperature or on ice to reduce the repair process) was observed in this study. We speculate that the characteristics of energy deposition from the x-ray irradiation (low linear energy transfer) may dictate the overall response of the cell towards viscosity and RI changes (the response of GFP alone to RI has been previously reported by us and others^17,28^. Manipulating the environment of DNA damaging probes and drugs, such as cisplatin, on viscosity may additionally provide important breakthroughs in cancer treatments including chemotherapy. Future studies may investigate the impact of other environmental factors such as pH and ionic strength on the lifetimes of these probes.

## Conclusion

The changes of intracellular and in particular nuclear viscosity have been monitored directly using fluorescence lifetime imaging and phosphorescence lifetime imaging microscopy, FLIM and PLIM. We demonstrate the first instance of using a phosphorescent metal complex, Pt(L)Cl, as a viscosity sensor in live cells using phosphorescence lifetime imaging. We further demonstrate a new application of fluorescent proteins, namely GFP (and derivatives), mCherry and mNeonGreen as fluorescent probes for measuring intracellular viscosity in live cells and in particular the nucleus (Table 1). A decrease in GFP emission lifetime and an increase in the lifetime of mCherry, mNeonGreen and especially in the phosphorescence lifetime of Pt[L]Cl with increasing viscosity illustrate the promise of these probes for monitoring viscosity changes in the course of intercellular processes. The lifetime of Pt[L]Cl is the most affected by changes in viscosity, offering a variation from 12 to 14 microseconds from 10 to 100 cP versus only 0.3 ns difference in fluorescent probes. This large effect of viscosity on the lifetime of the Pt[L]Cl, and the ease of detection by broadly available picosecond and nanosecond lifetime detection systems, makes it a better choice for the viscosity measurements in cells compared to the fluorescence protein probes. The new approach allowed us to detect directly changes in intracellular, and intranuclear, viscosity following DNA damage induced by a variety of means one-photon blue light, two-photon red light, or X-ray irradiation. The increase in viscosity following DNA damage is believed to be biomolecular condensate driven and caused by several factors including chromatin remodeling, the recruitment of DNA repair proteins and nuclear actin filament polymerization at the damage site. Overall, this work offers a new tool to measure intracellular viscosity using phosphorescent, long-lived probes – a particularly important development due to a very large number of emissive, cell-permeable transition complexes that became available in recent years. This new tool may open up a way for better understanding of cell damage and repair processes, particularly the mechanism and effects of DNA damage. It also opens a new perspective on processes that may be affected by gene function including therapy, drug action and reaction dynamics in the cytoplasm and nucleus.

**Table 1:** Summary of mean changes in environmental sensing probe.

Probe in cells	Emission Lifetime before and after irradiation inducing DNA damage	
	Before irradiation	After irradiation
GFP	2.65 ± 0.1 ns	2.29 ± 0.1 ns
mNeonGreen	2.8 ns (bacteria)	2.4 ± 0.1 ns
mCherry	1.54 ns ± 0.2 ns	1.93 ± 0.2 ns
PtLCl	12.5 ± 0.8 µs	14.1 ± 1.1 µs

## Supplementary Information

Supplementary Figures.

## Data Availability

All raw data are available upon request and will also be stored on UKRI-STFC server for 5–10 years. Please contact the corresponding author, Stan.Botchway@stfc.ac.uk.
