# Supplementary material for: Fluorescence and phosphorescence lifetime imaging reveals a significant cell nuclear viscosity and refractive index changes upon DNA damage
Source: Sci Rep. 2023 Jan 9;13:422. doi: 10.1038/s41598-022-26880-x (PMC9829731; doi:10.1038/s41598-022-26880-x)
Supplement: Supplementary file 1 — Supplementary Figures. [file 41598_2022_26880_MOESM1_ESM.docx]

**Supplementary Material**

# **Fluorescence and Phosphorescence Lifetime Imaging Reveals a Significant Cell Nuclear Viscosity and Refractive Index changes Increase Upon DNA Damage**

Ellen Clancy^1^, Siva Ramadurai^2^, Sarah R. Needham^1^, Julia Weinstein^3^, Karen Baker^4^, Tara A. Eastwood^4^, Dan Mulvihill^4^, Stanley W. Botchway^1*^

^1^Central Laser Facility, UKRI- Science and Technology Facilities Council, Rutherford Appleton Laboratory, Harwell Science and Innovation Campus, Oxfordshire, OX11 0QX, U.K.

^2^The Rosalind Franklin Institute, Rutherford Appleton Laboratory Harwell Campus, Didcot, OX11 0QX

^3^Department of Chemistry, Dainton Building, 13 Brook Hill, Sheffield, S3 7HF, UK

^4^School of Biosciences, University of Kent, Canterbury, Kent, CT2 7NJ, UK

*Corresponding Author: E-mail: [Stan.Botchway@stfc.ac.uk](mailto:Stan.Botchway@stfc.ac.uk)

**Supplementary Material**


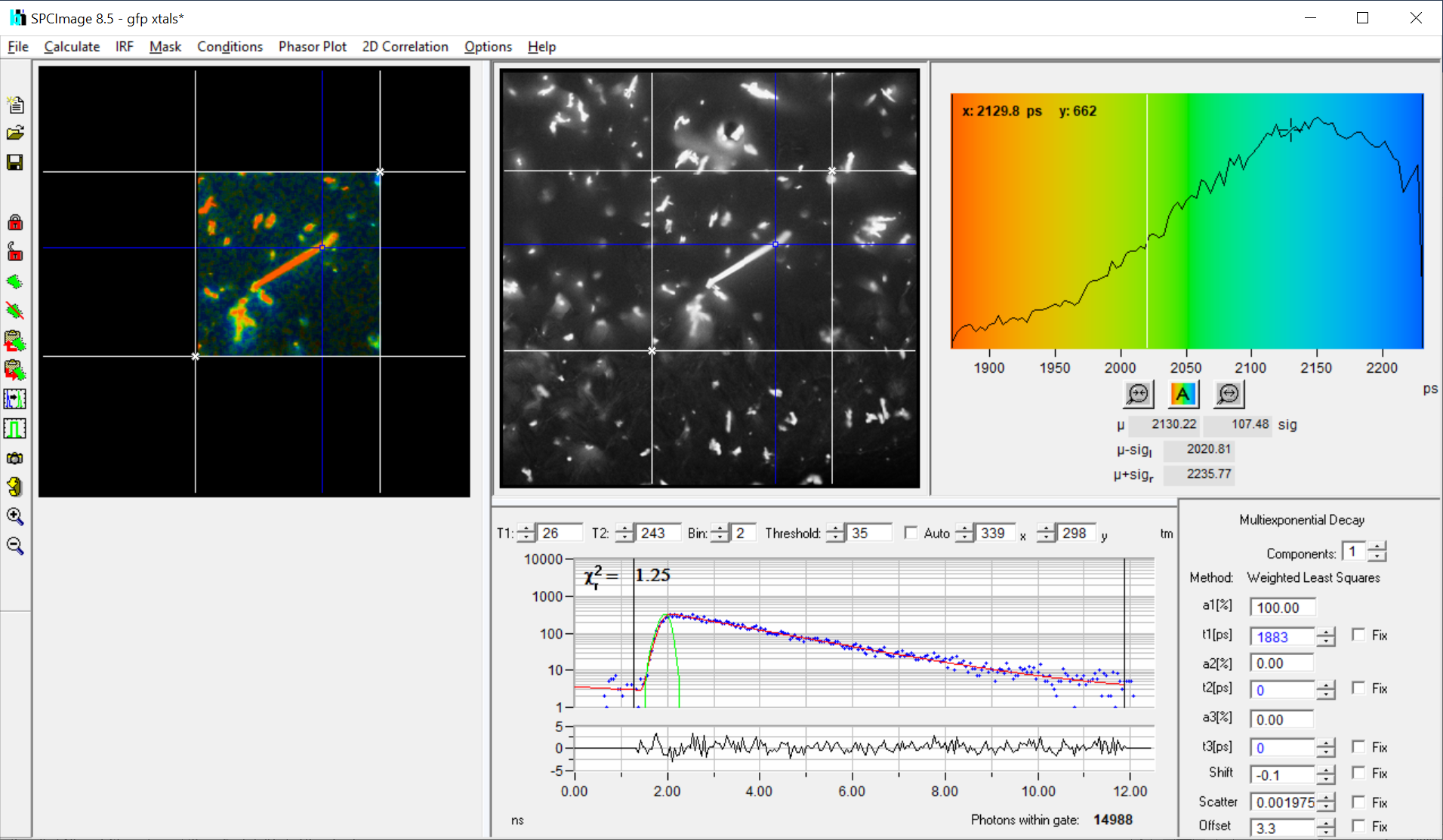


***Fig. S1a.*** *GFP micro-crystals. Significantly reduced lifetimes are observed when GFP is locked in a crystalline state. A lifetime range of 1.650-2.2 ns was observed compared with GFP in solution of around 2.5-2.7 ns.*

Fig. S1b

EGFP fluorescence lifetime (inverse τ^-1^) versus refractive index (RI squared) of water:glycerol mixture indicating a general increasing trend consistent with that observed with RI, satisfying the Strickler-Berg relationship. Points on graph are the average of 3 experiments with errors less than 10% calculated as standard error on the mean.

S2a

S2b

**Fig. S2** – Fluorescence lifetime of mCherry is lengthened with increasing viscosity **(a)** Semilogarithmic plot of the fluorescence decay of 1.5 µM mCherry in mixtures of glycerol. The fluorescence decays shown are representative of three measurements. **(b)** Semilogarithmic plot of the fluorescence lifetime of mCherry in varying viscosities made by varying glycerol concentration. Standard deviation plotted as error bars

S3a

S3b

**Fig. S3** – Fluorescence lifetime of mNeon Green is shortened with increasing viscosity **(a)** Semilogarithmic plot of the fluorescence decay of 16 µM mNeonGreen in mixtures of glycerol. The fluorescence decays shown are representative of three measurements. **(b)** Semilogarithmic plot of the fluorescence lifetime of mNeonGreen in varying viscosities made by varying glycerol concentration. Three repeated experiments. Standard deviation plotted as error bars

**Fig. S4** – Fluorescence lifetime of GFP is shortened with increasing temperature. Graph illustrating the fluorescence lifetime of GFP at varying temperatures.Three repeated experiments with error bars presented as the SD.

**Fig. S5** –Phosphorescence lifetime of Pt[L]Cl is not affected by increasing temperature. Graph illustrating the phosphorescence lifetime of Pt[L]Cl at varying temperatures. Three repeated experiments with error bars presented as the SD.

**Fig. S6** – Phosphorescene lifetime of Pt[L]Cl in various sucrose/water and purging conditions


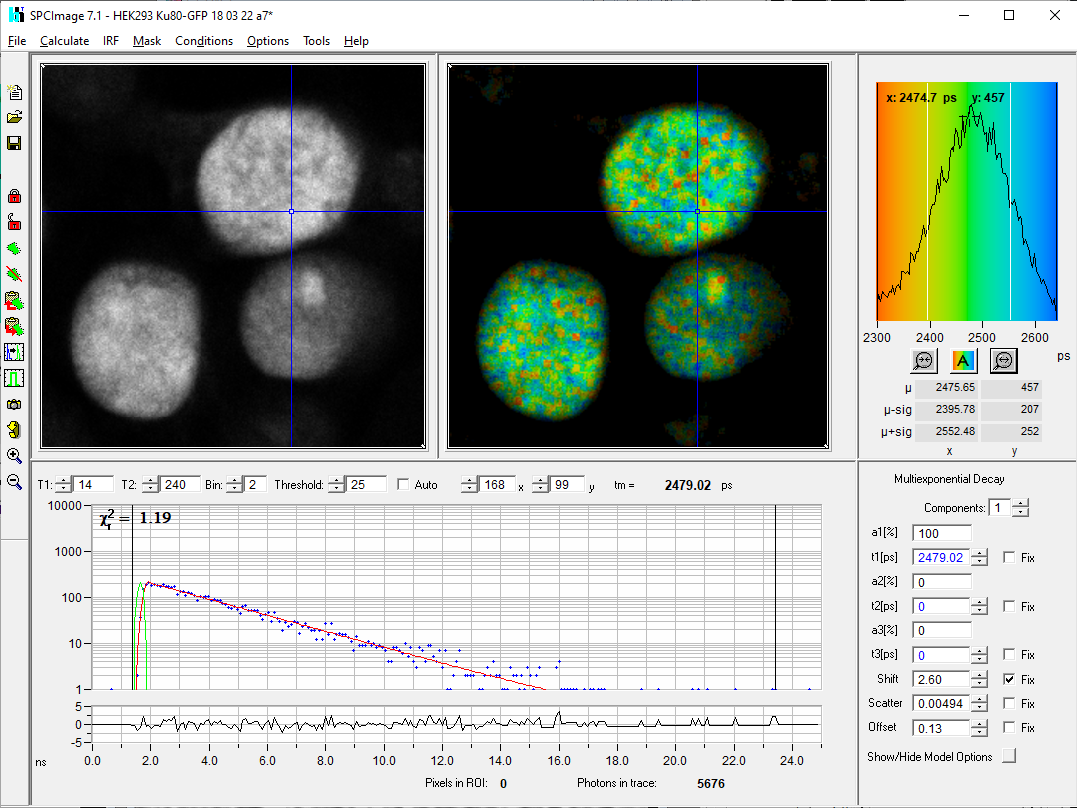

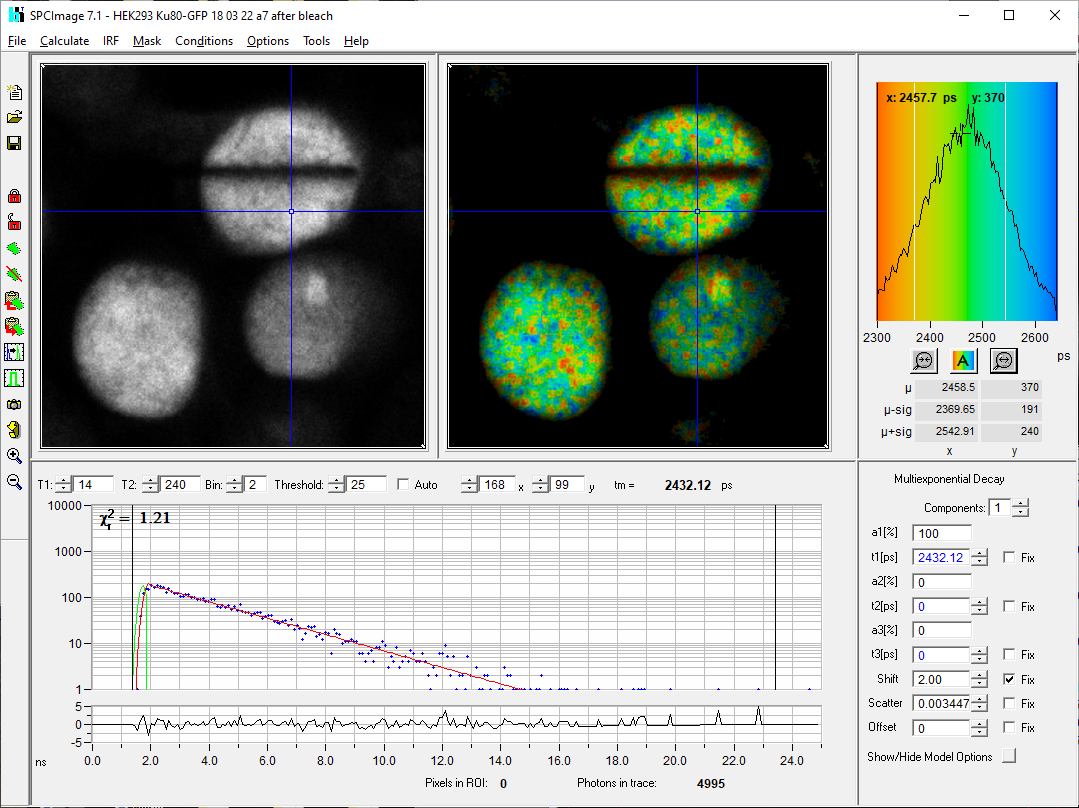


**Fig. S7** – Lifetime of fixed CHO cells expressing GFP-Ku80 did not change upon significant photo damage (20uW for 10 sec). FoV 30 um
